# Acute Effect of Normobaric Hypoxia on Performance in Repeated Wingate Tests with Longer Recovery Periods and Neuromuscular Fatigue in Triathletes: Sex Differences

**DOI:** 10.3390/jfmk10030282

**Published:** 2025-07-22

**Authors:** Víctor Toro-Román, Pol Simón-Sánchez, Víctor Illera-Domínguez, Carla Pérez-Chirinos, Sara González-Millán, Lluís Albesa-Albiol, Sara Ledesma, Vinyet Solé, Oriol Teruel, Bruno Fernández-Valdés

**Affiliations:** 1 Research Group in Technology Applied to High Performance and Health, Department of Health Sciences, TecnoCampus, Universitat Pompeu Fabra, Mataró, 08302 Barcelona, Spain; vtoro@tecnocampus.cat (V.T.-R.); psimon@edu.tecnocampus.cat (P.S.-S.); sgonzalezm@tecnocampus.cat (S.G.-M.); lalbesa@tecnocampus.cat (L.A.-A.); sledesma@edu.tecnocampus.cat (S.L.); vsole@edu.tecnocampus.cat (V.S.); oteruelg@edu.tecnocampus.cat (O.T.); bfernandez-valdes@tecnocampus.cat (B.F.-V.); 2 Faculty of Communications and Social Sciences, University of San Jorge, Villanueva de Gállego, 50830 Zaragoza, Spain

**Keywords:** cycloergometer, repeat-sprint ability, power, oxygen, vertical jump

## Abstract

**Background**: Repeated high-intensity intervals under normoxic (NOR) and hypoxic (HYP) conditions is a training strategy used by athletes. Although different protocols have been used, the effect of longer recovery between repetitions is unclear. In addition, information on the effect of repeated high-intensity intervals on HYP in women is scarce. **Aims**: To analyse the differences between sexes and between conditions (NOR and HYP) in Repeated Wingate (RW) performance and neuromuscular fatigue in triathletes. **Methods**: A total of 12 triathletes (men: *n* = 7, 23.00 ± 4.04 years; women: *n* = 5, 20.40 ± 3.91) participated in this randomised, blinded, crossover study. In two separate sessions over seven days, participants performed 3 × 30” all out with 7′ of recovery in randomised NOR (fraction of inspired oxygen: ≈20%; ≈300 m altitude) and HYP (fraction of inspired oxygen: ≈15.5%; ≈2500 m altitude) conditions. Before and after RW, vertical jump tests were performed to assess neuromuscular fatigue. Oxygen saturation, power, perceived exertion, muscle soreness and heart rate parameters were assessed. **Results**: Significant differences were reported between sexes in the parameters of vertical jump, oxygen saturation, RW performance and heart rate (*p* < 0.05). However, between conditions (NOR and HYP), only differences in oxygen saturation were reported (*p* < 0.05). No significant differences were reported between conditions (NOR and HYP) in RW performance, neuromuscular fatigue, muscle soreness and perception of exertion. **Conclusions**: A 3 × 30” RW protocol with 7′ recovery in HYP could have no negative consequences on performance, neuromuscular fatigue and perception of exertion in triathletes compared to NOR, independently of sex.

## 1. Introduction

Triathlon is an endurance sport that combines swimming, cycling and running over different distances [1]. Specifically, during the cycling sector, large groups break to improve their position. These breaks occur with efforts at intensities close to the maximal oxygen uptake (VO_2_ max) [2]. Given the above, it is key that the athlete can produce short, intense efforts separated by recovery periods, which has been termed repeated-sprint ability (RSA) [3,4].

The Wingate test is a comprehensive 30-s ergometric test in which the athlete pedals against a resistance set according to a percentage of their body weight [5]. Repeated Wingates (RWs) are one method of RSA training [6], being a valid training strategy [7]. Although most studies using RW have been performed under normoxic conditions (NOR) [8,9,10], the implementation of this protocol under hypoxic conditions (HYP) has also been studied [11,12,13,14], using different training protocols: 6 × 30” with 2′ recovery [7], 3 × 30” with 4.5′ recovery [12,14] or 4 × 30” with 4.5′ recovery [11].

The reduced oxygen concentration in the HYP condition results in energy supply limitations, metabolite accumulation and impaired muscle contraction, among others [15,16]. It is known that RSA protocols in HYP conditions increase heart rate (HR), minute ventilation, oxygen debt, muscle deoxygenation and lactate, compared to NOR conditions [17,18]. Similarly, performing RSA in an HYP environment is likely to cause significant neuromuscular fatigue compared to NOR conditions [18,19].

Previous studies indicated that HYP does not affect the performance of a Wingate test [20,21]. Regarding RW, Takei et al. (2020) reported no significant differences in performance after performing 3 × 30” RW with 4.5′ recovery under HYP and NOR conditions [14]. Also, Takei et al. (2021) reported that, despite increased arterial hypoxaemia, a 4 × 30” RW protocol with 4.5′ recovery had no effect on performance and metabolic and neuromuscular adjustments [11].

Several studies have explored how different recovery durations affect RSA, employing diverse protocols. Among these, 30-s recovery intervals are the most used [9]. The decrease in energy production during RSA is influenced by the exercise–rest ratio [22]. Previous studies observed lower power loss at exercise–rest ratios of 1:3 compared to 1:1 and 1:2, in HYP and NOR conditions [23]. This suggests that with a lower exercise-to-rest ratio, the additional impact of hypoxia on RSA is reduced [23].

Various studies have analysed and described the acute and chronic changes in physical exercise under HYP conditions in recent years [24,25]. However, despite the large amount of the literature on specific responses to HYP, most studies were conducted in men [24,26]. In the review by Girard et al. (2017), within future lines of research, they highlighted that further research was needed to analyse sex differences during RSA [17]. The acute effect of HYP on RSA in women is scarce [23,27,28], and studies that differentiate between sexes are even scarcer. Men and women show anthropometric and physiological differences, which may make them respond differently to exercise-induced metabolic stress [29,30]. While sex differences in physiological responses have been described in NOR, it is not clear in the HYP condition [24,31].

Although there are recent studies showing that acute HYP exposure does not compromise performance during long repeated efforts with almost complete recoveries [11,12,14], information on the effect of longer recoveries is scarce. Therefore, the present study has two objectives: (i) to analyse the influence of HYP on performance and neuromuscular fatigue after an RW protocol with prolonged recoveries; (ii) to analyse sex differences in performance and neuromuscular fatigue after an RW protocol under HYP and NOR conditions. We hypothesised the following: (i) HYP exposure will decrease performance in an RW protocol and increase neuromuscular fatigue compared to the HYP condition; (ii) performance will be lower in female athletes compared to male athletes in both HYP and NOR conditions; (iii) performance will be lower in female athletes compared to male athletes in both HYP and NOR conditions; (iv) performance will be lower in male athletes compared to male athletes in both HYP and NOR conditions; and (v) performance will be lower in female athletes compared to male athletes in both HYP and NOR conditions.

## 2. Materials and Methods

### 2.1. Study Design

A randomised, crossover, blinded study was conducted. The research lasted 15 days. Each participant completed two experimental sessions, separated by one week to reduce training adaptations. All assessments were conducted in the afternoon and in the same order to avoid bias due to circadian rhythm interference. Randomisation was conducted using a website (www.randomizer.org).

Each participant performed two vertical jump tests before and after performing RW (3 × 30” all out) in different conditions: NOR (fraction of inspired oxygen [FiO_2_] ≈ 20%; ≈300 m altitude) and HYP (FiO_2_ ≈ 15.5%; ≈2500 m altitude). Oxygen (O_2_) concentration in the hypoxia chamber, pulse oxygen saturation (SpO_2_) and HR were monitored during repetitions and recovery. Subjective perception of exertion (RPE) and muscle soreness were also recorded.

To avoid interpretation by the participants, both assessments were performed in the hypoxia chamber (mean temperature before the start of the test 26.4 °C; mean temperature after the end of the test 27.4 °C; 65% relative humidity). Participants were instructed to avoid ergogenic supplements. All tests were conducted at an altitude of ~20 m above sea level.

**Figure 1:**
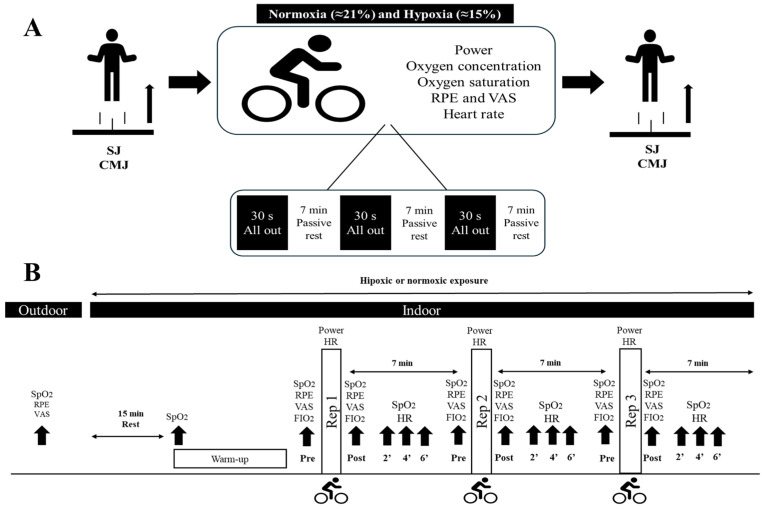
Study design. (**A**): General study protocol. (**B**): Timing of collection of the different parameters analysed during the protocol. HR: heart rate; SJ: squat jump; CMJ: countermovement jump; RPE: rating of perceived exertion; VAS: visual analogy scale; SpO_2_: pulse oxygen saturation.

Participants performed a warm-up on a cycloergometer at free intensity for 7′. Afterwards, they performed 5 repetitions of squats with vertical jump. Inside the chamber, after 15′ at rest, they performed a 5′ warm-up on the cycloergometer where they performed the test. Figure 1B shows the different assessments depending on the parameter to be evaluated.

### 2.2. Participants

**Figure 2:**
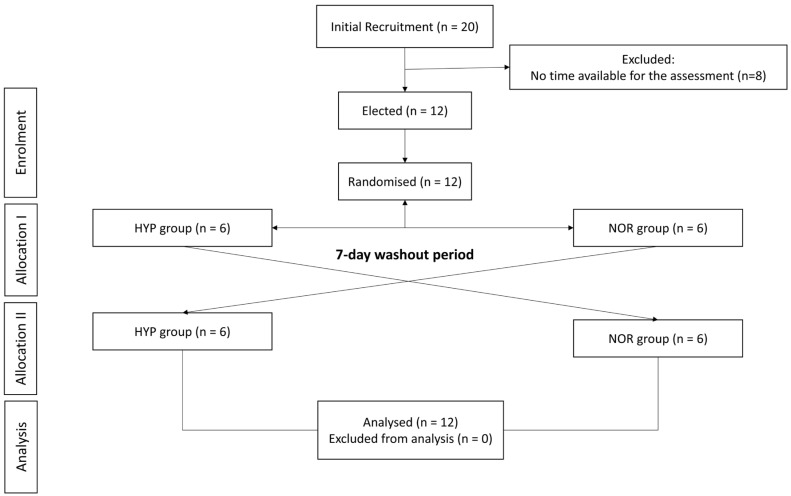
Study design flow-chart. HYP: hypoxia; NOR: normoxia.

An initial recruitment was carried out in different triathlon teams of the first Catalan division in the province of Barcelona. From the recruitment, 20 participants were interested. However, due to time incompatibility, 8 participants did not start the study (Figure 2). A total of 12 triathletes (men: *n* = 7; women: *n* = 5) participated in the present study. The characteristics of the subjects are shown in Table 1. All participants were triathletes of the Catalan League, 10 of them competing at national or international level, lived near sea level (<200 m) and had not been exposed to hypoxic environments in the 6 months prior to the study. After sample recruitment, post hoc analysis of the sample size was performed. The 12 participants represented a statistical power of 0.64 for an effect size of 0.5 and an alpha error of 0.05.

All participants were informed about the purpose of the study and signed a consent form before enrolment. The research design was approved by the TecnoCampus (Universitat Pompeu Fabra) ethics committee (approval number: 1/2024). The study was conducted in accordance with local legislation and institutional requirements. Each participant was assigned a code at sample collection and processing to maintain anonymity. Participants were encouraged to reduce the volume and intensity of their training two days prior to the assessments. Also, the intake of 6–8 g/kg/day of carbohydrate in the 48 h prior to the assessments was recommended [32]. During the signing of the informed consent, participants completed a form on general training and performance characteristics (Table 1).

To be included in the study, participants had to meet a number of criteria: (i) 18–30 years of age; (ii) be federated; (iii) 3 years of experience competing in triathlon; (iv) not suffer from any type of injury or illness that reduces or limits performance; (v) not consume medications or drugs; (vi) not have trained under hypoxia or at high altitude in the last 6 months; (vii) not consume sports supplements that may influence muscle oxygenation or neuromuscular fatigue during the study; (viii) have trained regularly, without significant injury, during the last 3 months.

### 2.3. Altitude Simulation

All tests (NOR and HYP) were performed in the hypoxic chamber (King Size resting chamber; 212 cm long × 200 cm wide × 160 cm high, Biolaster, Guipúzcoa, Spain). The chamber was connected to a hypoxic generator (Hypoxic Summit II, Biolaster, Spain; 38 cm × 48 cm base and 68 cm height; minimum flow rate 60 L/min). Under NOR conditions, to avoid subject interpretation, the generator was activated at the lowest possible altitude (simulated theoretical altitude ≈ 150 m). In HYP conditions, the generator was activated at a simulated theoretical altitude of ≈3000 m. As shown in the figure, the actual altitude reached was ≈2500 m (FiO_2_ ≈ 15.5%). The %FiO_2_ was monitored with an oxygen sensor (R-17VAN, Biolaster; Spain) calibrated before the titrations. In all conditions, only one person per test was allowed inside the chamber to avoid excess carbon dioxide (CO_2_). NOR-HYP blinding of participants was achieved by covering all monitor screens.

**Figure 3:**
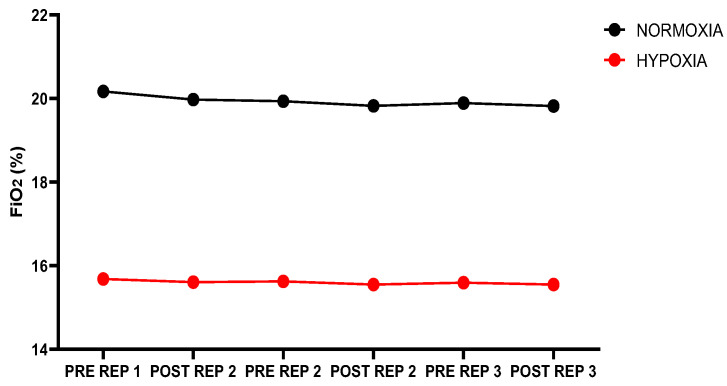
Inspired oxygen fraction in different conditions; FIO_2_: inspired oxygen fraction; REP: repetition.

For HYP and NOR, participants started inhaling chamber air 15 min before the specific warm-up. Figure 3 shows %FiO_2_ before (PRE) and after (POST) each repetition (REP) under different conditions (NOR and HYP).

### 2.4. Oxygen Saturation

SpO_2_ was continuously monitored with two devices placed on the index finger, one on each hand (Nonin WristOx2™, Model 3150, Plymouth, MN, USA). The sensor was attached to the left index finger and held at the wrist to ensure that it did not move during sprinting. Recording was performed at different times during the assessments: outside the chamber, inside the chamber (post 15′ inside), before the start of each high-intensity repetition, just after each high-intensity repetition and at 2′, 4′ and 6′ of recovery (Figure 1). The highest value of the two devices was selected for analysis.

### 2.5. Neuromuscular Assessment

Neuromuscular performance was analysed using the vertical jump test. Two repetitions of Squat Jump (SJ) and countermovement jump (CMJ) were performed before and after (1 min) WR. For the SJ, participants began the movement with their knees bent at a 90° angle and their hands placed on their hips, then executed the vertical jump with maximum effort. For the execution of the CMJ, the participants started the action from an upright position with their hands resting on their waists. Maintaining the position, participants performed a knee flexion–extension followed by a jump of maximum possible intensity. A contact platform (Chronojump Boscosystems, Barcelona, Spain) was used and jump height and flight time were recorded. Two repetitions of SJ and CMJ were performed, with a 30-s rest between jumps. The best jump was selected for analysis. Jump tests are quick and easy to perform, and many of the methods have been scientifically validated [33].

### 2.6. Repeated Wingate Protocol

Before starting the RW protocol, participants were seated for 15 min in the hypoxia chamber. They then completed a 5-min warm-up by cycling at low intensity (≈100 W).

The RW protocol consisted of 3 × 30” all out alternating with 7′ passive recovery periods. The workload during the all-out sets was 7.5% of each participant’s body weight. A cycloergometer (SOFT CARDGIRUS PRO1.2 3D; Cardgirus, Barcelona, Spain) was used for the tests. Data were recorded for maximum power output (PPO), mean power output (MPO) and fatigue index (FI) for each sprint. Seat positions were individually adjusted and repeated for each session. Both the sprints and the recovery phases were performed seated on the cycloergometer. Participants were encouraged during the sprints to maintain maximum intensity. Also, the cycloergometer monitor was covered during the sprints. Participants performed the test wearing their own running shoes.

### 2.7. Heart Rate, RPE and Muscle Soreness

HR was monitored at a frequency of 1 Hz using a chest strap sensor (Polar H10, Polar, Kempele, Finland). Pre-exercise HR was lowest during 15′ of rest in the hypoxia chamber. HRmax (maximum HR) was the maximum obtained in each repetition of 30′ all out. Regarding recovery, the mean HR was recorded during the 7′. Similarly, at rest, before and after each repetition, participants assessed their RPE (Borg scale 6–20) where 6 was defined as ‘very, very light’ effort and 20 as ‘maximum, strenuous’ effort [34] and muscle soreness by visual analogy scale (VAS) (0–10 scale) where 0 was defined as ‘minimal soreness’ and 10 as ‘maximal soreness’. These methods of perceptual assessment have been used previously [35].

### 2.8. Statistical Analysis

Statistical analysis was performed with IBM^®^ SPSS^®^ Statistics version 22 (IBM Corp., Armonk, NY, USA). The Shapiro–Wilk test was used to determine the normality of the data. Three-factor and two-factor (sex, condition and time) Analysis of Variance (ANOVA) tests were performed. Bonferroni post hoc was also applied for the time factor. Figure 6 shows the ANOVA statistical analysis for each repetition. On the other hand, for the percentage change in neuromuscular fatigue, a Student’s t-test for independent samples was performed. The effect size was calculated using partial eta squared (ηp^2^), where 0.01–0.06, 0.06–0.14 and >0.14 were considered small, moderate and large effect sizes, respectively [36]. The value of *p* < 0.05 was established as a statistically significant difference.

## 3. Results

The following section presents the results obtained in the study. Table 1 shows the participants’ characteristics. Significant sex differences in weight, height, better running time and better cycling power values were reported (*p* < 0.05).

Table 2 shows the results obtained from the vertical jump tests. Significant differences were observed between sexes across all analysed parameters (*p* < 0.01). However, no differences were found between conditions (NOR and HYP), nor in the percentage change between sexes.

In the SJ test, the following statistical results were obtained: (i) Flight time: sex (*p* < 0.001; ηp^2^ = 0.706), time (*p* = 0.747; ηp^2^ = 0.003), condition (*p* = 0.237; ηp^2^ = 0.035), sex × time (*p* = 0.765; ηp^2^ = 0.002), sex × condition (*p* = 0.827; ηp^2^ = 0.001), time × condition (*p* = 0.992; ηp^2^ = 0.000), sex × time × condition (*p* = 0.768; ηp^2^ = 0.002). (ii) Jump height: sex (*p* < 0.001; ηp^2^ = 710), time (*p* = 0.710; ηp^2^ = 0.003), condition (*p* = 0.250; ηp^2^ = 0.033), sex × time (*p* = 0.790; ηp^2^ = 0.002), sex × condition (*p* = 0.946; ηp^2^ = 0.000), time × condition (*p* = 0.967; ηp^2^ = 0.000), sex × time × condition (*p* = 0.779; ηp^2^ = 0.000).

For the CMJ test, the following results were reported: (i) Flight time: sex (*p* < 0.001; ηp^2^ = 0.697), time (*p* = 0.272; ηp^2^ = 0.030), condition (*p* = 0.197; ηp^2^ = 0.041), sex × time (*p* = 0.838; ηp^2^ = 0.697), sex × condition (*p* = 0.886; ηp^2^ = 0.001), time × condition (*p* = 0.878; ηp^2^ = 0.001), sex × time × condition (*p* = 0.717; ηp^2^ = 0.003). (ii) Jump height: sex (*p* < 0.001; ηp^2^ = 0.704), time (*p* = 0.253; ηp^2^ = 0.033), condition (*p* = 0.197; ηp^2^ = 0.041), sex × time (*p* = 0.838; ηp^2^ = 0.002), sex × condition (*p* = 0.957; ηp^2^ = 0.000), time × condition (*p* = 0.834; ηp^2^ = 0.001), sex × time × condition (*p* = 0.685; ηp^2^ = 0.004).

**Figure 4:**
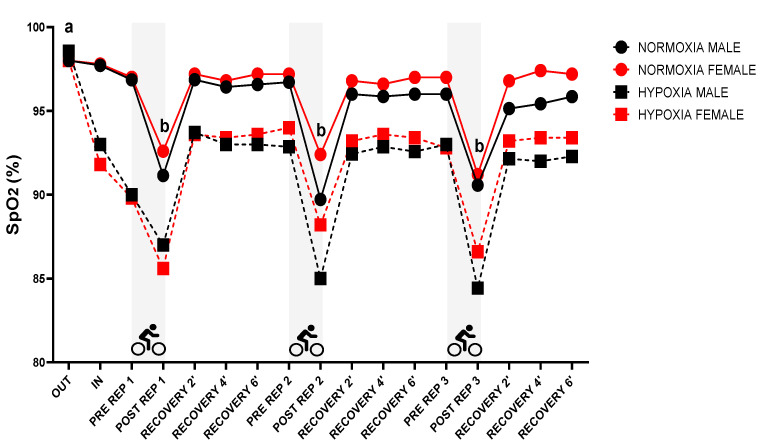
Pulse oxygen saturation during protocol; SpO_2_: pulse oxygen saturation; REP: repetition; a: *p* < 0.05 differences vs. other assessments; b: *p* < 0.05 differences vs. other assessments.

Figure 4 shows SpO_2_ during the protocol. Significant differences were found for sex (*p* = 0.001; ηp^2^ = 0.049), condition (*p* < 0.001; ηp^2^ = 0.572) and time (*p* < 0.001; ηp^2^ = 0.703). Specifically, resting values outside the hypoxic chamber (OUT) differed significantly from all other time points (*p* < 0.05). Likewise, post-repetition values differed significantly from the remaining time points (*p* < 0.05). Regarding interaction effects, a significant condition × time interaction was found (*p* < 0.001; ηp^2^ = 0.166). No other interactions were significant: sex × condition (*p* = 0.728; ηp^2^ = 0.003), sex × time (*p* = 0.272; ηp^2^ = 0.058), sex × condition × time (*p* = 0.970; ηp^2^ = 0.018).

**Figure 5:**
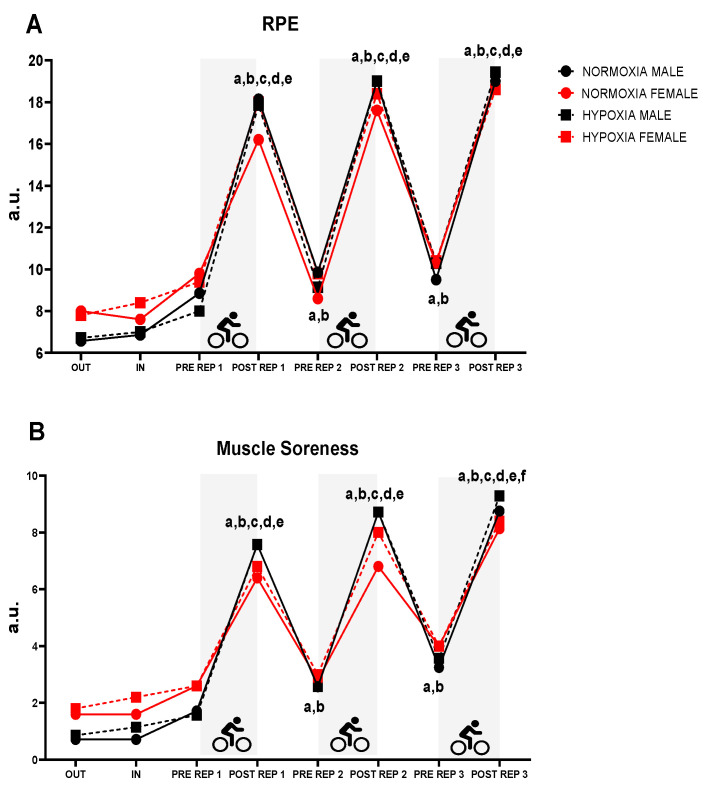
(**A**): RPE; (**B**): muscle soreness; RPE: rating of perceived exertion; REP: repetition; a: *p* < 0.05 differences vs. OUT; b: *p* < 0.05 differences vs. IN; c: *p* < 0.05 differences vs. PRE REP 1; d: *p* < 0.05 differences vs. PRE REP 2; e: *p* < 0.05 differences vs. PRE REP 3; f: *p* < 0.05 differences vs. POST REP 1.

Figure 5 presents the data of RPE and muscle soreness throughout the protocol. Significant differences over time were reported for both RPE (*p* < 0.001; ηp^2^ = 0.904) and muscle soreness (*p* < 0.01; ηp^2^ = 0.831). These differences were observed between OUT, IN, before repetitions (PRE REP 1, 2 and 3) and after repetition 1 (POST REP 1). A significant sex × time interaction was also found for muscle soreness (*p* = 0.014; ηp^2^ = 0.130).

For RPE, the following results were obtained: sex (*p* = 0.532; ηp^2^ = 0.004), condition (*p* = 0.382; ηp^2^ = 0.006), sex × condition (*p* = 0.293; ηp^2^ = 0.024), sex × time (*p* = 0.101; ηp^2^ = 0.092), condition × time (*p* = 0.917; ηp^2^ = 0.016) and sex × condition × time (*p* = 0.776; ηp^2^ = 0.014).

For muscle soreness: sex (*p* = 0.862; ηp^2^ = 0.006), condition (*p* = 0.323; ηp^2^ = 0.009), sex × condition (*p* = 0.464; ηp^2^ = 0.022), condition × time (*p* = 0.964; ηp^2^ = 0.016) and sex × condition × time (*p* = 0.979; ηp^2^ = 0.014).

**Figure 6:**
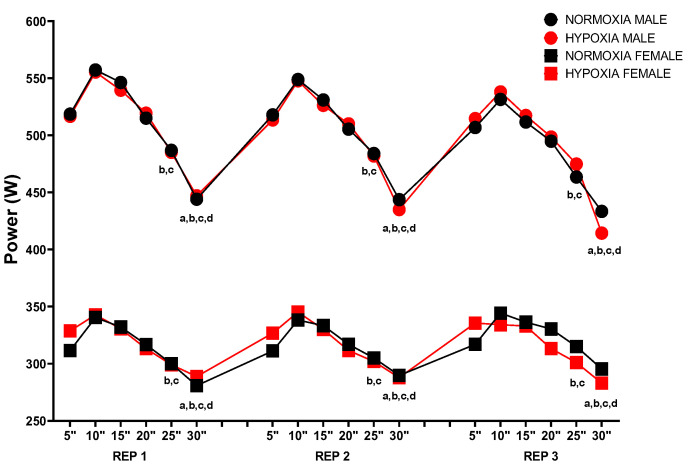
Mean power output every 5”; a: *p* < 0.05 differences vs. 5”; b: *p* < 0.05 differences vs. 10”; c: *p* < 0.05 differences vs. 15”; d: *p* < 0.05 differences vs. 20”.

Figure 6 displays the MPO every 5 s during each repetition. (i) Repetition 1: sex (*p* < 0.001; ηp^2^ = 0.821), condition (*p* = 0.866; ηp^2^ = 0.000), time (*p* < 0.001; ηp^2^ = 0.270), sex × condition (*p* = 0.793; ηp^2^ = 0.001), sex × time (*p* = 0.457; ηp^2^ = 0.038), condition × time (*p* = 0.999; ηp^2^ = 0.002), sex × condition × time (*p* = 0.999; ηp^2^ = 0.002). (ii) Repetition 2: sex (*p* < 0.001; ηp^2^ = 0.834), condition (*p* = 0.926; ηp^2^ = 0.000), time (*p* < 0.001; ηp^2^ = 0.280), sex × condition (*p* = 0.570; ηp^2^ = 0.001), sex × time (*p* = 0.305; ηp^2^ = 0.046), condition × time (*p* = 0.941; ηp^2^ = 0.002), sex × condition × time (*p* = 0.980; ηp^2^ = 0.003). (iii) Repetition 3: sex (*p* < 0.001; ηp^2^ = 0.799), condition (*p* = 0.823; ηp^2^ = 0.000), time (*p* < 0.001; ηp^2^ = 0.274), sex × condition (*p* = 0.570; ηp^2^ = 0.003), sex × time (*p* = 0.305; ηp^2^ = 0.048), condition × time (*p* = 0.941; ηp^2^ = 0.010), sex × condition × time (*p* = 0.980; ηp^2^ = 0.006).

According to the Bonferroni post hoc analysis, significant differences were found between the MPO values at 5”, 10”, 15” and 20” compared to 25” and 30” (*p* < 0.05).

Table 3 shows the PPO, MPO and FI values for each repetition performed during the protocol. Only differences between sexes were reported in all the parameters analysed (*p* < 0.01).

In PPO, the following statistics were obtained: sex (*p* < 0.001; ηp^2^ = 0.806), time (*p* = 0.865; ηp^2^ = 0.005), condition (*p* = 0.840; ηp^2^ = 0.001), sex*time (*p* = 0.837; ηp^2^ = 0.006), sex*condition (*p* = 0.974; ηp^2^ = 0.000), time*condition (*p* = 0.977; ηp^2^ = 0.001), sex*time*condition (*p* = 0.981; ηp^2^ = 0.001). In MPO, the following statistics were reported: sex (*p* < 0.001; ηp^2^ = 0.846), time (*p* = 0.841; ηp^2^ = 0.006), condition (*p* = 0.966; ηp^2^ = 0.000), sex*time (*p* = 0.629; ηp^2^ = 0.015), sex*condition (*p* = 0.981; ηp^2^ = 0.000), time*condition (*p* = 0.991; ηp^2^ = 0.000), sex*time*condition (*p* = 0.950; ηp^2^ = 0.002). In FI, the following statistics were reported: sex (*p* = 0.014; ηp^2^ = 0.097), time (*p* = 0.897; ηp^2^ = 0.004), condition (*p* = 0.302; ηp^2^ = 0.018), sex*time (*p* = 0.845; ηp^2^ = 0.006), sex*condition (*p* = 0.845; ηp^2^ = 0.001), time*condition (*p* = 0.273; ηp^2^ = 0.042), sex*time*condition (*p* = 0.992; ηp^2^ = 0.000).

Finally, Table 4 presents the data obtained during the HR protocol. There were significant differences between sexes in all parameters analysed (*p* < 0.001; ηp^2^ = 0.218). In addition, in HRmean, significant differences were reported in the time factor (*p* < 0.001; ηp^2^ = 624) and in the sex*condition interaction (*p* < 0.05; ηp^2^ = 0.052).

In HRrest, the following statistics were obtained: condition (*p* = 0.534; ηp^2^ = 0.020), sex*condition (*p* = 0.636; ηp^2^ = 0.011). In HRmax, the following statistics were obtained: sex (*p* < 0.001; ηp^2^ = 0.256), time (*p* = 0.763; ηp^2^ = 0.009), condition (*p* = 0.735; ηp^2^ = 0.002), sex*time (*p* = 0.927; ηp^2^ = 0.003), sex*condition (*p* = 0.735; ηp^2^ = 0.002), time*condition (*p* = 0.970; ηp^2^ = 0.001), sex*time*condition (*p* = 0.966; ηp^2^ = 0.001). In HRmean, the following statistics were obtained: condition (*p* = 0.602; ηp^2^ = 0.002), sex*time (*p* = 0.919; ηp^2^ = 0.012), time*condition (*p* = 0.966; ηp^2^ = 0.008), sex*time*condition (*p* = 0.882; ηp^2^ = 0.014).

## 4. Discussion

The aims of the present investigation were (i) to analyse the influence of HYP on performance and neuromuscular fatigue after an RW protocol with prolonged recoveries; (ii) to analyse sex differences in performance and neuromuscular fatigue after an RW protocol under HYP and NOR conditions. It was observed that the RW protocol (3 × 30” all out with 7′ passive recovery) performed under HYP did not negatively affect performance, neuromuscular fatigue or perceived exertion in participants compared to NOR, regardless of sex. Performance and HR were lower in female triathletes in both conditions. Previous research has demonstrated that HYP does not impair performance during RW. In relation to RW, Takei et al. (2020) found no significant differences in performance following a 3 × 30” RW protocol with 4.5′ of recovery under HYP and NOR conditions [14]. Furthermore, Takei et al. (2021) reported that a 4 × 30” RW protocol with 4.5′ of recovery did not affect performance [11].

To our knowledge, this is one of the first investigations to examine sex differences in performance and neuromuscular fatigue after an RW protocol with full recovery under HYP and NOR conditions. Previous research, such as that of Maldonado-Rodriguez et al. (2022), included two girls and five boys, but did not analyse sex differences [37]. On the other hand, the study by Piperi et al. (2024), although analysing sex differences, compared the effect of a training programme on HYP and not the acute effect [31]. Also, Camacho-Cardenosa et al. (2022) evaluated sex differences in cardiorespiratory responses to acute resting exposure in normobaric HYP compared to NOR [38].

Recovery type and duration between sprint repetitions significantly influence the capacity to generate and preserve PPO during repeated efforts, which is essential in numerous athletic disciplines [39]. In line with the results obtained in the present investigation, a 4 × 30” protocol with 4.5′ of recovery in HYP, despite causing greater arterial hypoxaemia and HR responses, did not significantly affect performance, muscle oxygenation and neuromuscular capacity compared to NOR [11]. Also, a 3 × 30” protocol with 4.5’ recovery had no negative consequences on performance. In addition, the lactate and RPE values were similar compared to NOR [14].

The similarity in RW performance under NOR and HYP conditions may, at least partially, be explained by the length of the recovery intervals between bouts. Two primary factors limit RSA performance: the efficiency of adenosine triphosphate (ATP) resynthesis relative to its utilisation, and the regulation of ionic imbalances induced by repeated high-intensity efforts [40]. After a maximal sprint of 6 s, muscle phosphocreatine (PCr) decreases to 35–55% of the resting level and recovers to 69% after 30” of rest [41]. In addition, non-oxidative glycolysis also decreases in successive sprints. Therefore, during successive sprints, there is a decrease in ATP resynthesis from PCr hydrolysis and non-oxidative glycolysis, leading to an increase in the oxidative contribution to ATP formation [18]. Indeed, a 4′ recovery period enhances total oxygen uptake [42], facilitating more complete PCr resynthesis, promoting muscle lactate oxidation and H^+^ efflux and supporting the reestablishment of ionic homeostasis—all of which are considered contributing factors to fatigue mitigation [40]. Previously, it was reported in men that a recovery period equal to or longer than 2 min could allow the progressive incorporation of aerobic metabolism during the recovery period as well as in subsequent repetitions [43]. In addition, increasing the rest time between efforts resulted in an improvement in the anaerobic contribution to repeated exercise [43]. Therefore, the 7′ of recovery is more than enough time for PCr resynthesis via oxidative energy pathways.

During recovery in RSA protocols, oxidative energy metabolism is importantly involved in restoring homeostasis through PCr resynthesis [44,45]. Sustaining high performance levels is largely linked to the resynthesis of PCr, a process that is strongly influenced by the length of the recovery period [46]. To achieve optimal RW implementation, modifying the oxidative–glycolytic balance by manipulating programming variables, such as the exercise–rest ratio of the severity of hypoxia during the session, can result in specific acute physiological and performance responses [22,47]. The magnitude of the decrease in performance, in turn, depends on the characteristics of the task (degree of HYP, number of repetitions, structure of the sprint series, etc.) [11]. It cannot be ruled out that greater fatigability would have been observed if more repetitions had been completed, if recovery between workouts had been shorter and/or if more severe levels of hypoxia had been tested [14].

Sex-related differences in RW performance are well documented, with men typically demonstrating higher absolute and relative power outputs than women across RSA [48]. This is likely due to men possessing greater muscle mass—both in total and relative to body weight—while women generally have a higher percentage of body fat, which is associated with reduced PPO during sprints [49]. Additionally, differences in muscle fibre types and metabolic profiles, both of which are influenced by sex, further contribute to performance decline [48]. Generally, men have a larger muscle fibre cross-sectional area and a proportionally larger area of type II muscle fibres, which translates into greater force generation [50]. However, type II fibres are more dependent on the anaerobic glycolytic pathway, which induces greater fatigability and slower recovery. In both conditions and groups, the oxygen deficit decreased from the first to the second sprint in each protocol. However, the recovery period did not significantly impair power during RW.

Decreased oxygen availability has been shown to have a detrimental effect on performance [40,51,52]. As exercise intensity increases, there is progressive muscle deoxygenation [51,53]. In the present study, in general, women showed higher SpO_2_ values compared to men. Previous studies speculated that women are less sensitive to a reduction in O_2_ availability [48,54]. Previously, it has been reported that the tissue saturation index during the sprint phases of the RSA test appeared to be higher in women than in men, both before and after the test [31]. These differences may be partly due to sex-specific variations in muscle fibre composition, as type I fibres—which are more prevalent in women—are associated with greater muscle oxygenation during maximal sprint efforts [55]. Additionally, oestrogen seems to play a role in promoting pulmonary vasodilation [56].

The sex difference in SpO_2_ could be related to neuromuscular fatigue. Although no significant differences were observed, in general, women expressed a higher percentage change than men. Previously, an inverse relationship between SpO_2_ and neuromuscular activity was observed where men showed a greater attenuation of neuromuscular activity for SpO_2_ [48]. The lower impact of O_2_ availability in women may be the result of attenuated sympathetic neural outflow, possibly due to oestrogen [48], which reduced vasoconstriction, maintained muscle perfusion and prolonged sprint endurance. The greater predominance of type I muscle fibres in women is associated with a better vasodilator response and oxygenation, resulting in lower levels of neuromuscular fatigue and less impaired contractile function [31]. However, it is noteworthy that no differences in neuromuscular fatigue were observed between NOR and HYP, consistent with previous research [11,13].

Regarding HR, in general, a lower HRmean was observed in HYP compared to NOR. These results agree with those reported by Takei et al. (2024) [13]. This decrease in HR could be attributed to compensatory vasodilatation in HYP, where dilatation of arteries and arterioles could lead to a decrease in HR [57]. Regarding perceptual responses, no differences were observed between sex and conditions (NOR and HYP). Similarly, Takei et al. (2021) reported no significant differences between HYP and NOR conditions in a 3 × 30” protocol with 4.5′ recovery [11]. It could be hypothesised that the affective response to repeated maximal effort cycling exercise is not influenced by systemic or local hypoxia [58], at least if the recovery periods between repetitions are prolonged.

### Limitations

The present study is not without limitations: (i) the menstrual cycle of the participants was not controlled. Some studies have hypothesised an increase in ventilatory levels and ultimately performance under hypoxic conditions during the luteal phase. However, there is no definitive answer [24]; (ii) although the sample is not large, 12 participants, with 5 girls in total, it is a representative sample compared to previous studies [13,14,37].

## 5. Conclusions

An RW protocol (3 × 30” all out with 7′ passive recovery) conducted under HYP did not negatively affect performance, neuromuscular fatigue or perceived exertion in triathletes compared to NOR, regardless of sex. However, female triathletes underperformed compared to their male triathletes in both HYP and NOR conditions. However, the SpO_2_ levels were higher in female triathletes.

## 6. Practical Applications

An RW protocol (3 × 30” all out with 7’ passive recovery) can be useful for maximum intensity training, in NOR and HYP conditions, without significantly decreasing training performance and without increasing neuromuscular fatigue and perceived exertion in both male and female triathletes. Coaches and athletes should be aware of the effects that recovery duration can have on physiological and performance responses in RW.

## Figures and Tables

**Table 1:** Participant characteristics.

		Total (*n* = 12)	Men (*n* = 7)	Women (*n* = 5)	*p*
**Age (years)**		21.91 ± 4.03	23.00 ± 4.04	20.40 ± 3.91	0.292
Weight (kg)		63.08 ± 8.06	67.57 ± 7.23	56.80 ± 3.96	0.013
Height (m)		1.71 ± 0.07	1.76 ± 0.04	1.64 ± 0.04	0.001
Competition level (*n*)	Regional	3	2	1	
	National	8	5	3	
	International	1	-	1	
Weekly training (last month)	Days	5.91 ± 1.50	6.14 ± 1.21	5.60 ± 1.94	0.563
	Hours	13.25 ± 5.89	13.42 ± 4.61	13.0 ± 7.96	0.908
Best time 5 km run race (min)		17.25 ± 1.70	16.06 ± 0.71	18.92 ± 1.12	<0.001
Best time 10 km run race (min)		37.07 ± 5.28	33.65 ± 1.67	42.20 ± 4.53	0.003
MPO obtained in 5′ test (W)		329.90 ± 57.13	357.00 ± 39.52	266.66 ± 37.85	0.010
MPO obtained in 10′ test (W)		275.50 ± 49.39	293.57 ± 41.37	233.33 ± 45.09	0.003

MPO: median power output.

**Table 2:** Flight time and jump height in SJ and CMJ tests.

	Sex	Time	NOR	HYP	S	T	C	S*T	S*C	T*C	S*T*C
Flight time SJ (s)	Male	Pre	0.472 ± 0.016	0.466 ± 0.020	**	NS	NS	NS	NS	NS	NS
		Post	0.469 ± 0.029	0.459 ± 0.024							
		%	−0.64	−1.49							
	Female	Pre	0.391 ± 0.033	0.377 ± 0.046							
		Post	0.388 ± 0.028	0.379 ± 0.031							
		%	−0.60	1.25							
Height SJ (s)	Male	Pre	27.37 ± 2.02	26.70 ± 2.33	**	NS	NS	NS	NS	NS	NS
		Post	27.10 ± 3.45	25.87 ± 2.80							
		%	−1.16	−2.51							
	Female	Pre	18.88 ± 3.18	17.60 ± 4.24							
		Post	18.58 ± 2.66	17.72 ± 2.81							
		%	−1.12	3.15							
Flight time CMJ (s)	Male	Pre	0.496 ± 0.181	0.490 ± 0.020	**	NS	NS	NS	NS	NS	NS
		Post	0.488 ± 0.036	0.472 ± 0.022							
		%	−1.48	−3.44							
	Female	Pre	0.408 ± 0.038	0.392 ± 0.050							
		Post	0.397 ± 0.029	0.385 ± 0.040							
		%	−2.31	−1.37							
Height CMJ (s)	Male	Pre	30.21 ± 2.17	29.47 ± 2.43	**	NS	NS	NS	NS	NS	NS
		Post	29.42 ± 4.39	27.44 ± 2.62							
		%	−2.48	−6.49							
	Female	Pre	20.55 ± 3.85	19.10 ± 4.90							
		Post	19.45 ± 2.83	18.92 ± 3.21							
		%	−4.28	−2.45							

SJ: Squat Jump; CMJ: countermovement jump; S: sex; T: time; C: conditions; NS: not significant; ** *p* < 0.01.

**Table 3:** PPO, MPO and FI during RW protocol.

	Sex	Time	NOR	HYP	S	T	C	S*T	S*C	T*C	S*T*C
PPO (W)	Male	REP 1	573.43 ± 58.67	572.71 ± 59.42	**	NS	NS	NS	NS	NS	NS
		REP 2	566.29 ± 66.49	565.86 ± 49.85							
		REP 3	563.05 ± 58.66	560.00 ± 48.64							
	Female	REP 1	354.40 ± 60.19	354.60 ± 54.59							
		REP 2	351.80 ± 53.61	355.80 ± 54.54							
		REP 3	354.00 ± 52.67	356.60 ± 46.05							
MPO (W)	Male	REP 1	508.00 ± 42.24	507.14 ± 43.41	**	NS	NS	NS	NS	NS	NS
		REP 2	501.86 ± 41.23	499.14 ± 38.48							
		REP 3	487.00 ± 43.89	490.00 ± 40.07							
	Female	REP 1	311.40 ± 43.38	315.20 ± 44.18							
		REP 2	314.40 ± 44.12	315.20 ± 43.55							
		REP 3	321.20 ± 43.35	314.60 ± 41.97							
FI (%)	Male	REP 1	22.17 ± 6.33	21.55 ± 6.38	*	NS	NS	NS	NS	NS	NS
		REP 2	21.24 ± 6.00	22.96 ± 6.02							
		REP 3	20.95 ± 6.74	25.64 ± 11.32							
	Female	REP 1	20.20 ± 4.60	18.31 ± 3.11							
		REP 2	17.24 ± 5.36	18.78 ± 4.88							
		REP 3	16.60 ± 2.23	20.68 ± 2.16							

NOR: normoxia; HYP: hypoxia; PPO: peak power output; MPO: mean power output; FI: fatigue index; S: sex; T: time; C: conditions; NS: not significant; ** *p* < 0.01; * *p* < 0.05.

**Table 4:** HR during protocol.

	Sex	Time	NOR	HYP	S	T	C	S*T	S*C	T*C	S*T*C
HRrest (bpm)	Male	-	70.86 ± 13.18	76.71 ± 15.45	**	-	NS	-	NS	-	-
	Female	-	65.60 ± 10.73	66.40 ± 8.62							
HRmax (bpm)	Male	REP 1	172.71 ± 7.43	170.71 ± 12.94	**	NS	NS	NS	NS	NS	NS
		REP 2	174.86 ± 7.19	172.29 ± 11.62							
		REP 3	174.14 ± 6.81	172.71 ± 10.16							
	Female	REP 1	157.00 ± 14.95	159.00 ± 15.68							
		REP 2	159.40 ± 14.82	158.60 ± 16.33							
		REP 3	162.20 ± 14.65	161.00 ± 16.20							
HRmean (bpm)	Male	REP 1	150.71 ± 14.09	146.00 ± 14.00	**	**	NS	NS	*	NS	NS
		Rec 1 ^	111.00 ± 11.83	101.14 ± 18.27							
		REP 2	156.71 ± 12.77	147.57 ± 13.37							
		Rec 2 ^	119.14 ± 10.73	109.86 ± 20.60							
		REP 3	156.57 ± 10.24	147.57 ± 22.97							
		Rec 3 ^	122.71 ± 11.23	110.00 ± 24.01							
	Female	REP 1	132.20 ± 15.27	137.40 ± 13.31							
		Rec 1 ^	84.40 ± 14.62	92.00 ± 23.06							
		REP 2	136.00 ± 18.98	132.20 ± 17.12							
		Rec 2 ^	89.20 ± 17.69	99.80 ± 25.94							
		REP 3	140.40 ± 10.31	141.00 ± 20.64							
		Rec 3 ^	86.80 ± 21.60	102.80 ± 27.16							

NOR: normoxia; HYP: hypoxia; HRrest: rest heart rate; HRmax: maximal heart rate; HRmean: mean heart rate; S: sex; T: time; C: conditions; NS: not significant; * *p* < 0.05; ** *p* < 0.01; ^: differences with respect to REP.

## Data Availability

The original contributions presented in the study are included in the article; further inquiries can be directed to the corresponding author.

## Abbreviations

The following abbreviations are used in this manuscript:

RW	Repeated Wingate
NOR	Normoxia
HYP	Hypoxia
FiO_2_	Fraction of inspired oxygen
O_2_	Oxygen
CO_2_	Carbon dioxide
RSA	Repeated-sprint ability
VO_2_ max	Maximal oxygen uptake
HR	Heart rate
CMJ	Countermovement jump
RPE	Rating of perceived exertion
VAS	Visual analogy scale
SpO_2_	Pulse oxygen saturation
MPO	Mean power output
PPO	Peak power output
FI	Fatigue index

## References

[1] Lepers R., Knechtle B., Stapley P.J. (2013). Trends in Triathlon Performance: Effects of Sex and Age. Sports Med..

[2] Bentley D.J., Millet G.P., Vleck V.E., McNaughton L.R. (2002). Specific Aspects of Contemporary Triathlon: Implications for Physiological Analysis and Performance. Sports Med..

[3] Gonzalez-Custodio A., Crespo C., Timón R., Olcina G. (2024). Effects of a Combined Method of Normobaric Hypoxia on the Repeated Sprint Ability Performance of a Nine-Time World Champion Triathlete: A Case Report. Behav. Sci..

[4] Bishop D., Girard O., Mendez-Villanueva A. (2011). Repeated-Sprint Ability—Part II: Recommendations for Training. Sports Med..

[5] Bar-Or O. (1987). The Wingate Anaerobic Test an Update on Methodology, Reliability and Validity. Sports Med..

[6] MacInnis M.J., Gibala M.J. (2017). Physiological Adaptations to Interval Training and the Role of Exercise Intensity. J. Physiol..

[7] Breenfeldt Andersen A., Bejder J., Bonne T., Olsen N.V., Nordsborg N. (2020). Repeated Wingate Sprints Is a Feasible High-Quality Training Strategy in Moderate Hypoxia. PLoS ONE.

[8] Buchheit M., Laursen P.B. (2013). High-Intensity Interval Training, Solutions to the Programming Puzzle: Part I: Cardiopulmonary Emphasis. Sports Med..

[9] Harbili S. (2015). The Effect of Different Recovery Duration on Repeated Anaerobic Performance in Elite Cyclists. J. Hum. Kinet..

[10] Lopez E.-I.D., Smoliga J.M., Zavorsky G.S. (2014). The Effect of Passive versus Active Recovery on Power Output over Six Repeated Wingate Sprints. Res. Q. Exerc. Sport.

[11] Takei N., Soo J., Hatta H., Girard O. (2021). Performance, Metabolic, and Neuromuscular Consequences of Repeated Wingates in Hypoxia and Normoxia: A Pilot Study. Int. J. Sports Physiol. Perform..

[12] Takei N., Kakinoki K., Girard O., Hatta H. (2020). Short-Term Repeated Wingate Training in Hypoxia and Normoxia in Sprinters. Front. Sports Act. Living.

[13] Takei N., Kakehata G., Inaba T., Morita Y., Sano H., Girard O., Hatta H. (2024). Effect of Hypoxic Sprint Interval Exercise and Normoxic Recovery on Performance and Acute Physiological Responses. Eur. J. Sport. Sci..

[14] Takei N., Kakinoki K., Girard O., Hatta H. (2020). No Influence of Acute Moderate Normobaric Hypoxia on Performance and Blood Lactate Concentration Responses to Repeated Wingates. Int. J. Sports Physiol. Perform..

[15] Powell F.L., Garcia N. (2000). Physiological Effects of Intermittent Hypoxia. High. Alt. Med. Biol..

[16] Kon M., Ohiwa N., Honda A., Matsubayashi T., Ikeda T., Akimoto T., Suzuki Y., Hirano Y., Russell A.P. (2014). Effects of Systemic Hypoxia on Human Muscular Adaptations to Resistance Exercise Training. Physiol. Rep..

[17] Girard O., Brocherie F., Millet G.P. (2017). Effects of Altitude/Hypoxia on Single-and Multiple-Sprint Performance: A Comprehensive Review. Sports Med..

[18] Bowtell J.L., Cooke K., Turner R., Mileva K.N., Sumners D.P. (2014). Acute Physiological and Performance Responses to Repeated Sprints in Varying Degrees of Hypoxia. J. Sci. Med. Sport..

[19] Racinais S., Bishop D., Denis R., Lattier G., Mendez-Villaneuva A., Perrey S. (2007). Muscle Deoxygenation and Neural Drive to the Muscle during Repeated Sprint Cycling. Med. Sci. Sports Exerc..

[20] McLellan T.M., Kavanagh M.F., Jacobs I. (1990). The Effect of Hypoxia on Performance during 30 s or 45 s of Supramaximal Exercise. Eur. J. Appl. Physiol. Occup. Physiol..

[21] Calbet J.A.L., De Paz J.A., Garatachea N., Cabeza de Vaca S., Chavarren J. (2003). Anaerobic Energy Provision Does Not Limit Wingate Exercise Performance in Endurance-Trained Cyclists. J. Appl. Physiol..

[22] Raberin A., Elmer J., Willis S., Richard T., Vernillo G., Iaia M., Girard O., Malatesta D., Millet G. (2022). The Oxidative-Glycolytic Balance Influenced by Sprint Duration Is Key during Repeated Sprints in Hypoxia. Med. Sci. Sports Exerc..

[23] Brosnan M.J., Martin D.T., Hahn A.G., Gore C.J., Hawley J.A. (2000). Impaired Interval Exercise Responses in Elite Female Cyclists at Moderate Simulated Altitude. J. Appl. Physiol..

[24] Raberin A., Burtscher J., Citherlet T., Manferdelli G., Krumm B., Bourdillon N., Antero J., Rasica L., Malatesta D., Brocherie F. (2023). Women at Altitude: Sex-Related Physiological Responses to Exercise in Hypoxia. Sports Med..

[25] Faiss R., Raberin A., Brocherie F., Millet G.P. (2024). Repeated-Sprint Training in Hypoxia: A Review with 10 Years of Perspective. J. Sports Sci..

[26] Cowley E.S., Olenick A.A., McNulty K.L., Ross E.Z. (2021). “Invisible Sportswomen”: The Sex Data Gap in Sport and Exercise Science Research. Women Sport. Phys. Act. J..

[27] Kasai N., Kojima C., Goto K. (2018). Metabolic and Performance Responses to Sprint Exercise under Hypoxia among Female Athletes. Sports Med. Int. Open.

[28] Bouten J., Brick M., Saboua A., Hadjadj J.-L., Piscione J., Margot C., Doucende G., Bourrel N., Millet G.P., Brocherie F. (2023). Effects of 2 Different Protocols of Repeated-Sprint Training in Hypoxia in Elite Female Rugby Sevens Players during an Altitude Training Camp. Int. J. Sports Physiol. Perform..

[29] Hunter S.K. (2016). The Relevance of Sex Differences in Performance Fatigability. Med. Sci. Sports Exerc..

[30] Santisteban K.J., Lovering A.T., Halliwill J.R., Minson C.T. (2022). Sex Differences in VO2max and the Impact on Endurance-Exercise Performance. Int. J. Environ. Res. Public Health.

[31] Piperi A., Warnier G., de Ten Ryen S.V.D., Benoit N., Antoine N., Copine S., Francaux M., Deldicque L. (2024). Repeated Sprint Training in Hypoxia Improves Repeated Sprint Ability to Exhaustion Similarly in Active Males and Females. Med. Sci. Sports Exerc..

[32] Vitale K., Getzin A. (2019). Nutrition and Supplement Update for the Endurance Athlete: Review and Recommendations. Nutrients.

[33] Alba-Jiménez C., Moreno-Doutres D., Peña J. (2022). Trends Assessing Neuromuscular Fatigue in Team Sports: A Narrative Review. Sports.

[34] Borg G. (1998). Borg’s Perceived Exertion and Pain Scales.

[35] Brocherie F., Millet G.P., Girard O. (2017). Psychophysiological Responses to Repeated-Sprint Training in Normobaric Hypoxia and Normoxia. Int. J. Sports Physiol. Perform..

[36] Hopkins W.G., Marshall S.W., Batterham A.M., Hanin J. (2009). Progressive Statistics for Studies in Sports Medicine and Exercise Science. Med. Sci. Sports Exerc..

[37] Maldonado-Rodriguez N., Bentley D.J., Logan-Sprenger H.M. (2022). Acute Physiological Response to Different Sprint Training Protocols in Normobaric Hypoxia. Int. J. Environ. Res. Public Health.

[38] Camacho-Cardenosa A., Camacho-Cardenosa M., Tomas-Carus P., Timón R., Olcina G., Burtscher M. (2022). Acute Physiological Response to a Normobaric Hypoxic Exposure: Sex Differences. Int. J. Biometeorol..

[39] Glaister M. (2005). Multiple Sprint Work: Physiological Responses, Mechanisms of Fatigue and the Influence of Aerobic Fitness. Sports Med..

[40] Girard O., Mendez-Villanueva A., Bishop D. (2011). Repeated-Sprint Ability—Part I: Factors Contributing to Fatigue. Sports Med..

[41] Gaitanos G.C., Williams C., Boobis L.H., Brooks S. (1993). Human Muscle Metabolism during Intermittent Maximal Exercise. J. Appl. Physiol..

[42] Haseler L.J., Hogan M.C., Richardson R.S. (1999). Skeletal Muscle Phosphocreatine Recovery in Exercise-Trained Humans Is Dependent on O2availability. J. Appl. Physiol..

[43] Feriche B., Delgado M., Calderón C., Lisbona O., Chirosa I.J., Miranda M.T., Fernandez J.M., Alvarez J. (2007). The Effect of Acute Moderate Hypoxia on Accumulated Oxygen Deficit during Intermittent Exercise in Nonacclimatized Men. J. Strength Cond. Res..

[44] Glaister M., Stone M.H., Stewart A.M., Hughes M., Moir G.L. (2005). The Influence of Recovery Duration on Multiple Sprint Cycling Performance. J. Strength Cond. Res..

[45] Selmi M.A., Sassi R.H., Yahmed M.H., Moalla W., Elloumi M. (2016). Effect of Between-Set Recovery Durations on Repeated Sprint Ability in Young Soccer Players. Biol. Sport..

[46] Dawson B., Goodman C., Lawrence S., Preen D., Polglaze T., Fitzsimons M., Fournier P. (1997). Muscle Phosphocreatine Repletion Following Single and Repeated Short Sprint Efforts. Scand. J. Med. Sci. Sports.

[47] Millet G.P., Faiss R. (2012). Hypoxic Conditions and Exercise-to-Rest Ratio Are Likely Paramount. Sports Med..

[48] Billaut F., Smith K. (2009). Sex Alters Impact of Repeated Bouts of Sprint Exercise on Neuromuscular Activity in Trained Athletes. Appl. Physiol. Nutr. Metab..

[49] Laurent C.M., Green J.M., Bishop P.A., Sjökvist J., Schumacker R.E., Richardson M.T., Curtner-Smith M. (2010). Effect of Gender on Fatigue and Recovery Following Maximal Intensity Repeated Sprint Performance. J. Sports Med. Phys. Fit..

[50] Miller A., MacDougall J.D., Tarnopolsky M.A., Sale D.G. (1993). Gender Differences in Strength and Muscle Fiber Characteristics. Eur. J. Appl. Physiol. Occup. Physiol..

[51] Willis S.J., Alvarez L., Millet G.P., Borrani F. (2017). Changes in Muscle and Cerebral Deoxygenation and Perfusion during Repeated Sprints in Hypoxia to Exhaustion. Front. Physiol..

[52] Billaut F., Buchheit M. (2013). Repeated-sprint Performance and Vastus Lateralis Oxygenation: Effect of Limited O 2 Availability. Scand. J. Med. Sci. Sports.

[53] Grassi B., Quaresima V., Marconi C., Ferrari M., Cerretelli P. (1999). Blood Lactate Accumulation and Muscle Deoxygenation during Incremental Exercise. J. Appl. Physiol..

[54] Fulco C.S., Rock P.B., Muza S.R., Lammi E., Braun B., Cymerman A., Moore L.G., Lewis S.F. (2001). Gender Alters Impact of Hypobaric Hypoxia on Adductor Pollicis Muscle Performance. J. Appl. Physiol..

[55] Kitada T., Machida S., Naito H. (2015). Influence of Muscle Fibre Composition on Muscle Oxygenation during Maximal Running. BMJ Open Sport Exerc. Med..

[56] Oliveira A.L.M.B., Rodrigues G.D., Silva B.M., de Azeredo Rohan P., da Silva Soares P.P. (2025). Sex Differences in Cardiorespiratory Control under Hypoxia: The Roles of Oxygen Desaturation and Hypoxic Exposure Time. Front. Cardiovasc. Med..

[57] Casey D.P., Joyner M.J. (2012). Compensatory Vasodilatation during Hypoxic Exercise: Mechanisms Responsible for Matching Oxygen Supply to Demand. J. Physiol..

[58] Behrendt T., Bielitzki R., Behrens M., Schega L. (2023). Acute Performance, Physiological, and Perceptual Changes in Response to Repeated Cycling Sprint Exercise Combined with Systemic and Local Hypoxia in Young Males. Physiol. Behav..

